# Advanced Technologies for the Improvement of Spray Application Techniques in Spanish Viticulture: An Overview

**DOI:** 10.3390/s140100691

**Published:** 2014-01-02

**Authors:** Emilio Gil, Jaume Arnó, Jordi Llorens, Ricardo Sanz, Jordi Llop, Joan R. Rosell-Polo, Montserrat Gallart, Alexandre Escolà

**Affiliations:** 1 Department of Agri Food Engineering and Biotechnology, Universitat Politècnica de Catalunya, Esteve Terradas 8, Campus del Baix Llobregat D4, 08860 Castelledfels, Barcelona, Spain; E-Mails: Jordi.Llorens.Calveras@upc.edu (J.L.); Jordi.Llop-Casamada@upc.edu (J.L.); Montserrat.Gallart@upc.edu (M.G.); 2 Department of Agricultural and Forest Engineering, Research Group in AgroICT & Precision Agriculture, Universitat de Lleida, Rovira Roure 191, 25198 Lleida, Catalonia, Spain; E-Mails: JArno@eagrof.udl.cat (J.A.); RSanz@eagrof.udl.cat (R.S.); JRRosell@eagrof.udl.cat (J.R.R.-P.); AEscola@eagrof.udl.cat (A.E.)

**Keywords:** vineyard, spraying techniques, precision viticulture, LIDAR, ultrasound

## Abstract

Spraying techniques have been undergoing continuous evolution in recent decades. This paper presents part of the research work carried out in Spain in the field of sensors for characterizing vineyard canopies and monitoring spray drift in order to improve vineyard spraying and make it more sustainable. Some methods and geostatistical procedures for mapping vineyard parameters are proposed, and the development of a variable rate sprayer is described. All these technologies are interesting in terms of adjusting the amount of pesticides applied to the target canopy.

## 1. Introduction

Crop protection is a key issue in farm management. It involves dealing with important risks and expensive pesticide products. Most of these products are to be applied by means of sprayers. Spraying techniques have been continuously evolving in recent decades. However, it is not only the sprayer itself, but everything related to the application of plant protection products (PPP) that needs to be taken into account in order to improve the results obtained. What is the canopy like? What is the canopy volume? What is the leaf area to be sprayed? What technologies are available to help growers improve their spray applications in a more effective, efficient and sustainable way?

In the 1980s, the term “precision agriculture” was used for the first time. Sensors, agricultural and statistical techniques and technology were combined to improve farm management techniques. One of the contexts where precision agriculture has been widely implemented is vineyards, in what is known as “precision viticulture”. After some decades of rapid development, in this day and age when people are carrying around extremely powerful microprocessors and GPS receivers in their pockets, it seems reasonable to consider using these technologies to turn spray applications into a more sustainable operation.

In the late 1990s, researchers from the *Universitat Politècnica de Catalunya* and the *Universitat de Lleida* focused their work on improving the spray application of PPP in vineyards and orchards. Some interesting results have been obtained with public funding coming from the Spanish Government and the coordination with other researchers from the Department of Agriculture of the Catalan Government, the *Universitat Politècnica de València* and the *Institut Valencià d'Investigacions Agràries*. In this paper, part of that work regarding the use of sensors to retrieve data from the vineyard canopies and to monitor the drift produced during the spray applications is reviewed. Some methods and geostatistical procedures for mapping vineyard parameters are proposed, and the development of a variable rate sprayer is described. After about two decades of development efforts, it is time now to put everything together and at the disposal of growers with the objective of improving the spray applications in Spanish viticulture.

## 2. Sensors for Canopy Characterization

The first step towards a crop-adapted application of PPP is the characterization of the vegetation to be treated. The information on geometrical and structural characteristics (height, width, volume, leaf density, leaf area, *etc.*) of plantations can help to optimize many agricultural tasks, such as irrigation and fertilization, as well as pruning and crop training techniques, among others. As far as pesticide applications is concerned, knowledge of the geometrical characteristics of the crop allows optimizing the dose of the applied product, and adjusting it to the characteristics of the plants, thereby obtaining a reduction of its environmental and economic impact [1].

**Figure 1.**
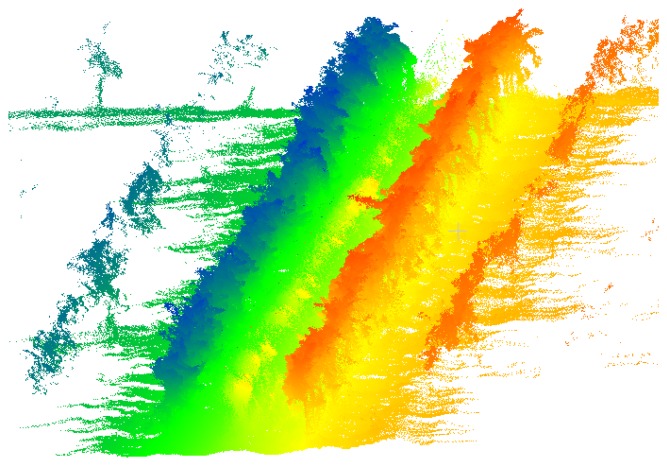
Point cloud obtained in a vineyard by the displacement of a LIDAR sensor along an alley between 2 rows (different colors correspond to different beam orientation angles).

Structural and geometric parameters of trees have been traditionally obtained using time-consuming and costly manual measurements, which are also destructive in the case leaves are removed to measure foliar surfaces. In recent years, different instruments and measurement systems for characterizing the canopy in a non-destructive, contactless, fast, accurate and repeatable way have appeared as alternatives to manual methods. The main systems used for the characterization of plants involve the use of electronic data acquisition systems associated with sensors based on different physical measuring principles. Other than ultrasound-based systems, most sensors are based on the use of electromagnetic radiation in certain spectral ranges or bands (visible, infrared, *etc.*). Ultrasonic sensors themselves, digital photography techniques, as well as stereoscopic vision and laser scanning sensors (Light Detection and Ranging—LIDAR scanners), are probably the most widely used and promising techniques for characterizing tree crops [1]. Among the above, both stereoscopic vision systems and LIDAR sensors stand out for the possibility of providing three-dimensional models of plantations [1–3]. Digital cameras are low-cost, easy-to-use and popular instruments suitable for estimating some plant characteristics such as height, volume and leaf area index (LAI) with reasonable accuracy. However, at present, they do not allow accurate 3D characterization of plantations in real time because of the complex and time-consuming post-processing algorithms required. Stereoscopic systems provide 3D models by combining two monocular images taken simultaneously with a binocular digital camera by means of computational algorithms. These systems lose effectiveness under certain environmental conditions, especially in variable lighting situations. Meanwhile, both ultrasonic sensors and LIDAR systems base their operation on the measurement of the distance between an emitter and a target object using pulsed sound waves or laser light, respectively. The distance is determined by measuring the time taken for the pulse to travel the distance from the emission point to the detection point after reflecting off the object (ultrasonic sensors and time-of-flight LIDAR) or, alternatively, by measuring the phase difference between the incident and the reflected waves (phase-shift LIDAR). The main disadvantages of ultrasonic sensors come from their low spatial resolution, highly divergent sonic cones, lack of scanning systems and potential interference between proximal sensors [4]. On the other hand, due to their high speed of measurement, resolution and accuracy, LIDAR systems are becoming one of the most used sensors for characterizing vegetation. If, in addition, the measurements of these sensors are synchronized with their spatial coordinates obtained by geo-referencing systems (e.g., GPS), it is possible to obtain maps of the parameters of interest of the plots analysed: canopy volumes, LAI, crop foliage density, 3D crop model, *etc*. Figure 1 shows a 3D point cloud obtained in a vineyard by displacing a LIDAR sensor along an alley between two adjacent rows.

However, it is still necessary to continue reducing the costs of sensors and associated electronics. It is also essential to develop suitable software programs for the post-processing of data, to increase both the usability and speed of calculation to assist decision making. This will contribute to the expansion of the use of systems for characterization of vegetation in many agricultural practices, being the application of pesticides one of which more advances and improvements may incorporate.

### 2.1. Measurement of Vegetative Volume

The use of terrestrial LIDAR technology for the geometric characterization of vineyards has mainly been concentrated in the last 10 years [2,3,5–9]. Two-dimensional terrestrial laser-LIDAR scanners (2D TLS) make two-dimensional sweeps in just one measuring plane. The additional third dimension can be obtained by moving the LIDAR in a direction perpendicular to the scanning plane. Although 2D TLS systems are normally simpler and more affordable than 3D TLS systems, they tend to be less accurate, and it can be difficult to properly control the movement of the LIDAR when collecting the data. Currently, the measurement systems based on 2D TLS allow three-dimensional scanning of vines with horizontal and vertical distances between points (size of the scan mesh) below 5 cm, and distance measurement accuracies of about 1 cm. To fully scan a row of vines, it is necessary to scan both sides of the row and unify both point clouds into a single coordinate system [3].

**Figure 2.**
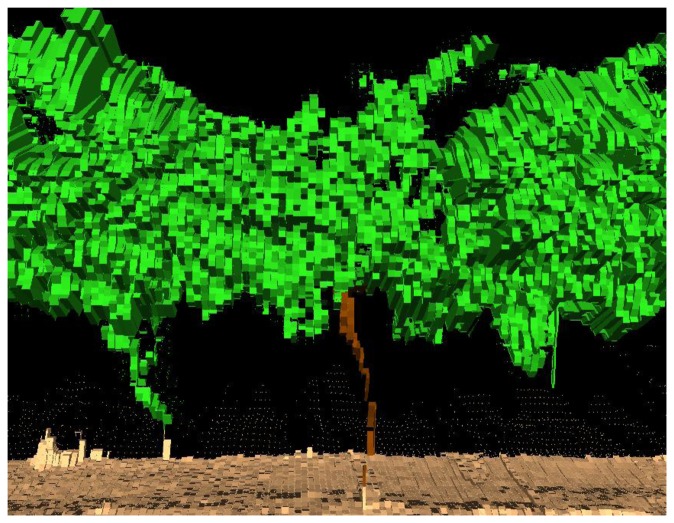
Resulting volume after processing the point cloud obtained with a 2D TLS dynamic measurement system in a vineyard.

The 3D point clouds obtained provide a huge amount of data. These, once processed into appropriately formatted information, can be very useful in the precise application of PPP. An important variable is the volume occupied by the point cloud (Figure 2), the so-called tree row LIDAR-volume (TRLV) [4]. The TRLV depends on: (i) the real size of the vegetation; (ii) the shape and size of the scan mesh; and (iii) the position(s) of the sensor with respect to the vineyard.

Since the main function of plants is photosynthesis, the distribution and position of the leaves is directly related to the availability of light. For this reason, the preferred position of leaves is normally on the outer layer of the crown. With this precedent, in [6] the hypothesis that there must be a non-linear relationship between the TRLV and the leaf area density (LAD) is presented.

For the calculation of the LAD in a section of the vineyard, one must know the TRLV and the leaf surface area. The latter is calculated by manually defoliating and measuring the surface of the leaves.

The study reported in [6] reveals a good logarithmic fit between the TRLV and the LAD. According to the results, the LAD can be estimated from the TRLV. If the LAD is multiplied by the TRLV, the leaf area of the vegetation under study can be obtained. It is therefore concluded that by using the information provided by the LIDAR 3D Dynamic Measurement System, a good estimation of the leaf area in hedgerow vineyards can be obtained.

Improvements in obtaining parameters related to geometric characterization such as TRLV, LAD or leaf area represent a major advance in improving the accurate application of PPP.

### 2.2. Leaf Area Index Estimation by Using the Tree Area Index Parameter

The LAI is defined as the one-side leaf area per unit of ground area and is probably the most widely used index for characterizing grapevine vigour. Currently, the LAI can be estimated using different types of sensors. Among the proposed technologies, vineyard leaf area can be indirectly estimated using ground-based laser sensors (or LIDAR systems) to obtain information about the geometry of the canopy [2,9,10]. Specifically, canopy volume is measured to subsequently obtain the total leaf area by an allometric relationship between both parameters. Faced with this procedure, [5] proposed to use the tree area index (TAI) to estimate the LAI in vineyards.

**Figure 3.**
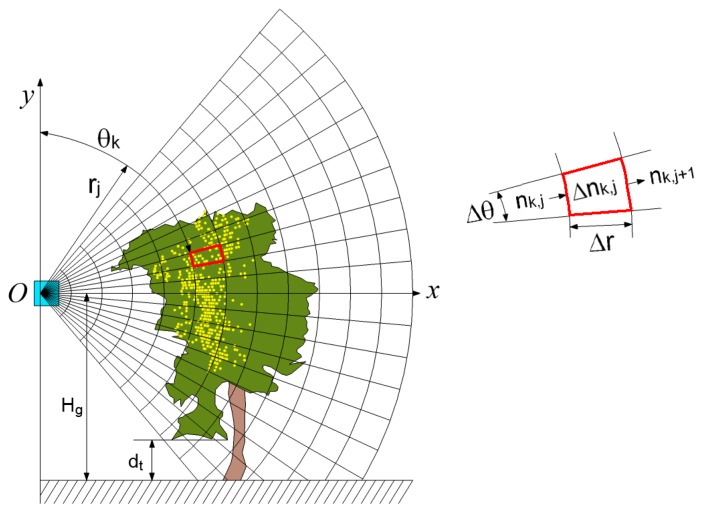
Two-dimensional grid of polar cells for calculating the tree area index Each polar cell is defined by two coordinates (*r_j_*, *θ_k_*). The first, *r_j_*, is the distance from the reference origin (LIDAR sensor), and the second, *θ_k_*, is the angle between the *Oy* axis and the radial direction (clockwise). *H_g_* is the height of the LIDAR sensor with respect to the ground (approximately constant, 1.65 m), and *d_t_* is the distance used to exclude intercepted points at ground and trunk level (with permission of the editor of *Precision Agriculture*).

The process for obtaining the TAI was explained in detail in [11] and was later adapted to vineyards by [5]. Adopting the reference system shown in Figure 3, the *Oz* axis (not shown) is parallel to the ground and in the direction in which the tractor-mounted LIDAR sensor moves. Thus, several scans can be obtained along the vine row, each of which results in a vertical semicircle in the plane *Oxy*. Finally, all interception points within the canopy are projected relative to the *Oz* axis onto a two-dimensional grid of polar cells in the *Oxy* plane (Figure 3). In particular, the overall projected cross-section of the canopy volume is divided into cells with equal angular increments of Δ*θ* = 3° and equal radial increments of Δ*r* = 100 mm. For each of the cells (*k*, *j*), it is possible to calculate the number of laser beams reaching the entrance side of the polar cell, *n_k,j_*, and the number of interceptions, Δ*n_k,j_*, within the cell. The TAI is finally calculated using Equation (1):

(1) $$\text{TAI}=-\frac{\Delta \theta }{W}\sum_{k=1}^{K}\sum_{j=1}^{J_{K}}r_{j}\delta_{k,j}\ln\left(1-\frac{\Delta n_{k,j}}{n_{k,j}}\right)$$

where, apart from the parameters Δ*θ*, Δ*n_k,j_* and *n_k,j_* described above, *W* (m) is the distance between the vine rows, *r_j_* is the radial distance between the polar cell and the sensor position and *δ_k,j_* is a binary variable that represents the presence or absence of foliage in each cell (*δ_k,j_* = 1 when the coefficient Δ*n_k,j_*/*n_k,j_* is greater than or equal to 0.01, and *δ_k,j_* = 0 when the coefficient is less than 0.01). In fact, the TAI is formulated as the ratio between the crop area detected by the LIDAR sensor and the ground area. In their calculation, it is also assumed that the probability of the laser beam's transmission within the vines could be approximated by the Poisson probability model when sufficiently small distances (Δ*r*) and a random spatial distribution of the leaves are considered. In any case, this approach allows the laser beam interception through the canopy to be described using an extinction probability model, in a manner similar to how the light extinction that occurs within vines is described.

To validate this method, the LMS-200 laser sensor (SICK AG, Waldkirch, Germany) has been used. More detailed information on this sensor can be found in [5]. The operation of the sensor is based on using the time-of-flight (TOF) principle to estimate the distances to the leaves within the canopy. The vines are scanned from one side of the row, and the data provided by the sensor are the polar coordinates of each interception point, *i.e.*, the radial distance (with an accuracy of ±15 mm in a single-shot measurement and 5 mm standard deviation in a range up to 8 m) and the angle of the laser beam (with an angular resolution of 1°). Data transfer from the sensor to a laptop is done via the RS-232 protocol using a MATLAB-based program for sensor control and data acquisition. The specific research conducted in a vineyard (cv. Merlot) in Raimat (Lleida, Spain) has made it possible to establish a model (Equation (2)) to estimate the leaf area using the TAI:

(2) LAI=1.2646TAI−0.1935

where *LAI* is the leaf area index (m^2^/m^2^) and *TAI* is the tree area index (dimensionless). The model can be applied to row lengths of 1 m, 2 m and 4 m, and has its main advantage in its applicability regardless of the side of the row from which the LIDAR reading is performed. Additionally, this method is non-destructive and does not require the use of allometric relationships. The disadvantages to this method are the large amount of data provided and the complexity of the algorithm used to calculate the TAI.

### 2.3. Electronic versus Manual Canopy Characterization

The accuracy of electronic measurements has been widely evaluated, and several field tests have been developed to compare electronic canopy estimations with manual measurements. The authors of [12] compared ultrasonic and laser measurements of citrus canopy volumes with manual measurement methods. They concluded that laser measurements provided a better prediction of canopy volume than the ultrasonic system because of the inherent higher resolution, but in any case they recommended the use of both ultrasonic and laser sensors for automatic mapping and quantification of the canopy volume of citrus trees. Arnó *et al.* used a LIDAR sensor to evaluate the leaf area index in vineyards, and the results were compared with manual measurements [13]. They found a good correlation between both values, which allowed the creation of canopy maps for subsequent applications. Based on that evidence, and with the aim to evaluate the different electronic alternatives for canopy characterization in vineyards, a comparative research study was carried out by [9]. The overall goal of that study was to evaluate the applicability of ultrasonic and LIDAR sensors for mapping canopy structures of different varieties and crop stages in vineyards, and to correlate the measurements of the canopy characteristics using manual methods, LIDAR and ultrasonic sensors.

Three different canopy parameters were manually measured in each field test: crop height, crop width and leaf area index. The measuring procedure was arranged according to [14], where the total canopy height was divided into three parts. For each of those parts, height and canopy width values were obtained. A partial leaf area corresponding to each of the three height levels was determined by applying the weight–area ratio obtained for every variety and crop stage. This ratio was determined by measuring the weight and surface area of 50 leaf samples collected from the bottom, middle and upper parts of the canopy in a randomized procedure, following the method described in [15]. The leaf surface (one side) was measured with a LI-COR LI 3100C electronic planimeter (LI-COR, Lincoln, NE, USA).

The obtained average values for the most important parameters used to define the canopy structure, such as crop height, crop width and crop volume obtained with the three investigated methods (manual, ultrasonic and LIDAR sensors) were analysed. A preliminary evaluation indicates that the values of canopy height measured manually (*C_HM_*) and with the LIDAR sensor (*C_HL_*) were relatively close. The values of crop width measured manually (*C_WM_*) were in all cases greater that those obtained with the ultrasonic sensor (*C_WU_*). The crop width obtained with the LIDAR sensor (*C_WL_*) results in the lowest values, probably due to it being the most precise scanning method and its greater ability to detect gaps in the canopy. Then, as a consequence of the observed tendency of those parameters, the measurements and estimations of crop volume present the same ranking, going from the highest values, obtained manually (*C_VM_*), to the lowest, obtained with the LIDAR sensor (*C_VL_*).

**Figure 4.**
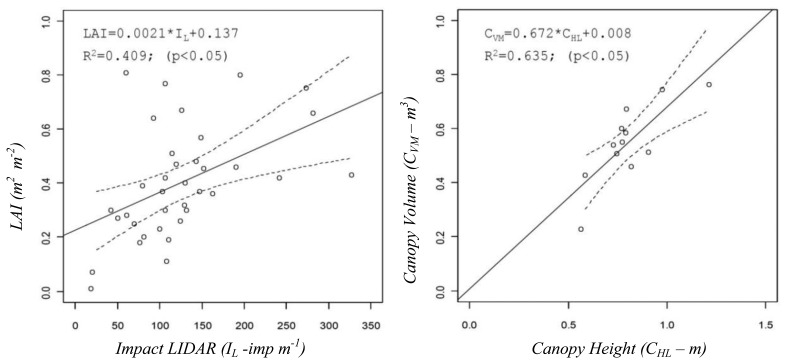
Relation between laser impacts obtained with LIDAR and LAI (**Left**). The correlation between canopy heights calculated with LIDAR and the canopy volume manually measured (**Right**) (published in [8], with permission of the editor of *Sensors* journal).

The comparative assessment included not only the above described and most important canopy structure characteristics but also others, obtained either from manual measurements in the field such as the leaf area index or numerical values derived from the use of the sensors, such as percentage of zero values measured on the crop with ultrasonic or LIDAR sensors, *Z_U_* and *Z_L_*, respectively, or *I_L_* (impacts·m^−1^) defined as the number of impacts (points where the laser beam detected the canopy). A good example of those relationships is shown in Figure 4, where the LAI can be predicted from the number of impacts obtained with LIDAR in the canopy or from the values of the canopy height measured with the same sensor, respectively.

The leaf wall area (*LWA*) is one of the proposed parameters to be used during pesticide spray applications in fruit crops, and is for that reason widely analysed in this field of research. Crop height values obtained with a LIDAR sensor (*C_HL_*) allow one to calculate the total leaf wall area on one side of a row canopy. The comparison of the manually estimated leaf wall area (*LWA_M_*) with the values measured with the LIDAR sensor (*LWA_L_*) indicates that in most cases the manual estimation of this parameter exceeds that obtained with the LIDAR sensor by about 30%, except in some particular cases. Those differences can be related to the total row length, *L*, with average values of 0.29 m^2^·m^−1^. Those differences can substantially affect the calculation of the total amount of pesticide applied to a target area, leading to unnecessary overdose.

One of the most important problems during the electronic process of canopy characterization using electronic devices comes from the tedious process of data analysis. In order to facilitate this process, a specific tool, PROTOLIDAR v1.0, was developed [16] to use data specifically from the LidarScan v.1^®^ program [17], with the aim of helping in the process of analysing the data generated by LIDAR measurements. PROTOLIDAR [18] was created to run in R^®^ and was released under the GPL 2 license. It consists of a package with a set of functions that helps to characterize the canopy of the grapevine (height, width and front view) from the LIDAR scan, performs statistical analysis on the outputs, plots and calculates the LAI, LWA and Tree Row Volume (TRV).

## 3. Vineyard Mapping

### 3.1. Mapping the Leaf Area Index Using A Ground-Based LIDAR Scanner

Precision viticulture (PV) is a concept that is beginning to have an impact on the wine-growing sector [19]. Grape-yield maps and remote sensing tools are used with varying success, the management of the spatial variability of grape quality being the remaining challenge. Furthermore, the application of plant protection products also raises controversy about the risk of contamination by drift and residue in grapes due to overdosing. The use of vegetation maps (LAI maps) might be an interesting way to assist in defining zones of different leaf surfaces. Then these zones could be managed differentially by applying the proper dose and avoiding environmental problems.

**Figure 5.**
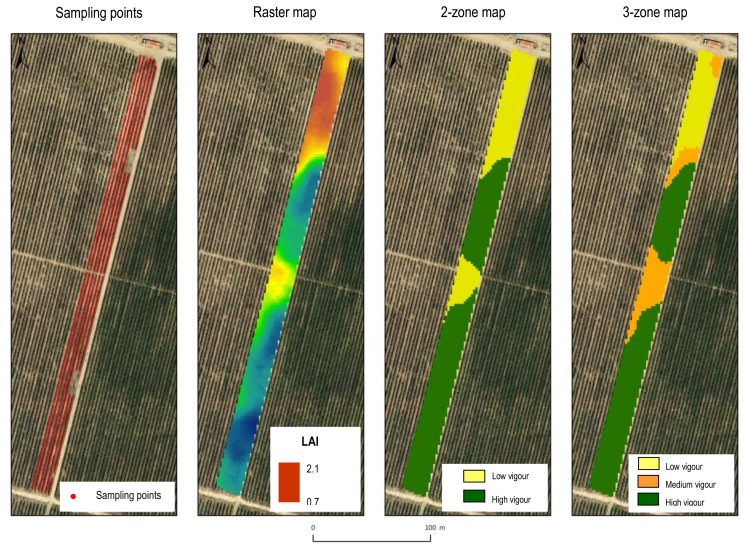
Sampling points, LAI raster map and maps of the LAI zones.

So far, there are few references in relation to obtaining vineyard LAI maps using LIDAR sensors. In any case, to facilitate the subsequent zoning according to leaf surface, it is advisable to generate LAI raster maps using a specific protocol associated with LIDAR sensor data. This idea has been discussed in [20], by mapping six rows of vines (cv. Syrah) in Raimat (Lleida, Spain), occupying an area of about 0.70 ha. The plot is ideal for generating a vegetation map due to the presence of a considerable spatial variation in the vine vigour along the rows, probably due to changes in topography within the plot. The LIDAR sensor used was again the SICK LMS-200, together with an inertial sensor (IMU, Inertial Measurement Unit) and a GX 1230 GG model GPS + RTK system (Leica Geosystems AG, St. Gallen, Switzerland) for later georeferencing and mapping the acquired information. Considering LIDAR readings from both sides of the row, the LAI per meter of row length can be estimated by calculating the left and right side areas (leaf wall areas including gaps) and the area which encloses the top of the row [2]. LAI values can then be georeferenced at points that are equally spaced by 1 m and are placed along the line of the grapevine trunks. The information generated (*X*, *Y*, *LAI*) can be displayed as a point vector map, and then converted to a raster map by geostatistical interpolation (Figure 5). Specifically, the raster map can be obtained by using the VESPER software [21], in this case, using a block kriging and a grid of 2 m for the projection of the interpolated data.

The spatial variability of the LAI is evident (Figure 5), and, more importantly, the pattern of variation seems to be highly structured, allowing the delimitation of well-defined and compact areas within the field. Adopting the approach suggested by [22], an unsupervised classification algorithm (fuzzy *c*-means) can be applied to the interpolated data to classify the LAI and then generate LAI zone maps (two or three zones, Figure 5). Ultimately, it is possible to optimize the applied doses of pesticides according to the amount of vegetation in each area and according to the benefits in product savings and drift reduction. There are also difficulties: The post processing is somewhat complex since the LIDAR sensor and GPS receiver normally have different frequencies, and the LIDAR data needs to be adjusted to match the LIDAR readings from both sides of the row.

### 3.2. Georeferenced LIDAR 3D Vine Plantation Map Generation

Generation of canopy maps and their further use in the improvement of different agronomy procedures in fruit and wine plantations has been the objective of several research groups. In the same way, canopy map generation has enhanced the quality of information obtained using LIDAR sensors, which has improved the knowledge of vegetation parameters. In addition, during the last decade, the systems for geographic positioning have helped to link all data obtained in the field into an accurate global geographic position. This work was conducted to generate a georeferenced canopy map of measured vine plantations using LIDAR measurements [8]. This process used to georeference every single point obtained with the LIDAR sensor is defined in [8]. Data obtained with LIDAR sensors while driving a tractor along a crop row can be managed and transformed into canopy impact density maps by evaluating the frequency of LIDAR returns. The readings have been made using the precise position information provided by a DGPS (Differential Global Positioning System) sensor.

**Figure 6.**
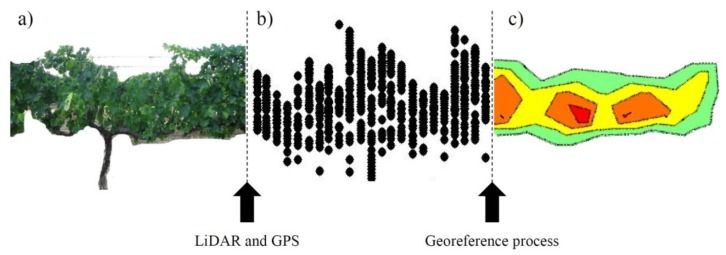
Graphical process to georeference LIDAR sensor data using the information provided by the DGPS system. The final results provide maps of the impact density in vineyards. (**a**) Image of real vines, lateral view; (**b**) Point cloud obtained, lateral view; (**c**) Impact density map generated, top view.

In that research, the team followed the same scan technique discussed in Section 2.3 of this paper, a technique that permits the scanning of the vineyard crop from both sides (Figure 6a). This scan provides a point cloud that defines the crop with high accuracy (Figure 6b). This point cloud can be georeferenced using the geographic position provided by the DGPS system; when this information is processed, it is possible to obtain an impact density map (Figure 6c). The impact density represents the foliar density of the canopy that has been scanned.

This methodology was applied and tested in different vine varieties, and the results show an accurate definition of some crop parameters; this is for the case of the LAI, which in its conventional implementation is a destructive measurement.

This same process of calculation can be useful to create maps of plantations using different sensors or analysing different parameters from this LIDAR sensor, like height, width or foliage density of the crop. These maps could be used either in real time to instantaneously modify the working parameters of the sprayer in sprayer applications, or in a post-process way as a base of information for DSS (decision support systems) tools. [13,23–25].

**Figure 7.**
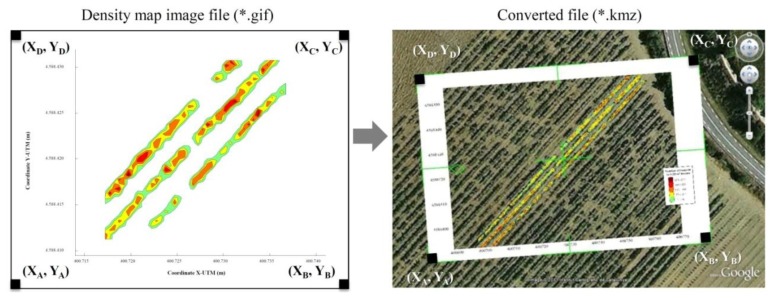
Procedure for conversion of an image file to a KMZ file. This proposed method superimposes the density map on the image of the field (published in [8] with permission of the editor of *Sensors* journal).

Another interesting step in the developed process was the process to share and visualize the obtained impact density maps. Once the impact density map was obtained, the UTM coordinates of each pixel were defined. Applying a conversion process to this image led to the production of a new file that was saved as a Google Earth^®^ compatible file (a KMZ file). The use of Virtual Globes, such as Google Earth^®^ and NASA World Wind representations of scientific data, have been thoroughly reviewed by [26]. Launching the KMZ file on a computer in which Google Earth^®^ had previously been installed enables the proposed application of the obtained maps and allows the drawing of the generated density map exactly over the orthophotograph of the measured parcel (Figure 7). Identification of gaps, leaf accumulation zones or other related aspects affecting canopy development can then be used in the crop management process.

## 4. Variable Rate Application of Plant Protection Products

Electronic canopy characterization allows the implementation of variable application rate techniques in fruit and vineyard crops, whereby pesticide application rates are modified according to crop characteristics [15,23,24,27–33]. In all cases, relevant benefits in terms of dose reduction, drift control and uniform deposition were achieved by all of the proposed methods. In the specific case of vineyards, the research group of *Universitat Politècnica de Catalunya* has developed a sprayer prototype that can apply a variable amount of liquid according to the canopy variability along the crop row [33]. The control algorithm is based on the measurement of the canopy width and its variations along the crop line. Once that parameter is electronically determined, information about the forward speed of the tractor along the row and the canopy height of every single measured section is added; the algorithm was developed in order to calculate the canopy volume to be sprayed per unit time, which is expressed in m^3^·min^−1^. Equation (3) indicates the relationship applied for this process:

(3) $$C_{Vj}=\frac{1,000}{60}\times C_{Wj}\times C_{Hj}\times \upsilon$$

where *C_Vj_* is the unit canopy volume to be sprayed per unit time at section *j* (m^3^·min^−1^); *C_Wj_*, the canopy width at a certain position at section *j* (m); *C_Hj_*, the canopy height at section *j* (m); and *v*, the forward speed of the tractor (km·h^−1^).

The main objective of the algorithm was to modify the emitted nozzle flow rate based on the measurements of the canopy volume along the crop row and its variations in order to maintain a constant objective application coefficient of 0.095 L·m^−3^, selected according to previous research [34,35]. The prototype was developed to be capable of a variable application rate according to the canopy variations along the crop line by proper modification of the nozzle flow rate.

In order to obtain continuous data about the canopy characteristics, three ultrasonic sensors were fitted to a stainless steel mast placed on the left side of a conventional air-blast orchard sprayer. A GPS antenna was also installed on top of the mast so that a GPS receiver could be used to evaluate the uniformity of the forward speed along the track and to record geographical coordinates. The sensors continuously estimated the canopy width from only the left side of the sprayer. All the sensors were connected to a controller placed in a waterproof box located on the right rear side of the sprayer. The controller was a Compact Field Point (National Instruments, Austin, TX, USA) equipped with analogue and digital input/output modules. A rugged computer and wireless router were also connected to remotely monitor and control the system. A box containing three sets of electrovalves (proportional and on–off), an electronic flow meter and a general pressure sensor were installed on top of the sprayer at the rear. Individual pressure sensors were also placed on every manifold.

The results indicated an average potential saving of 21.9%. There was a higher savings potential in the narrow canopy zones where the canopy width was below 0.22 m, which had an average savings of 31.4%. This value dropped to a 12.5% average for zones with a canopy width of over 0.22 m. These results indicated a similar response by the prototype that was independent of the canopy variation; instead, it was influenced by the crop stage and sensor position. In general, these estimated savings values are similar to the results of previous research [15,36] and can be directly related to more precise and safe use of plant protection products in accordance with the new European Directive for the sustainable use of pesticides [37].

## 5. Use of LIDAR Sensor in Drift Measurements

In general, the arrangement of field tests for drift measurement is very difficult and expensive. The ISO 22866:2005 standard defines the procedure to quantify drift during field tests, but this method is complex, time-consuming and heavily depends on external conditions such as wind speed and direction, which makes such tests difficult to use and may result in poor repeatability of results. As an alternative process, using sensor technology is another interesting option for drift evaluation purposes. Several studies [7,38–40] were carried out using LIDAR technology to measure drift. The specific scenario of spray processes in orchards is one of the most risky activities from the environmental point of view. In [7], a LIDAR system was used to measure the concentration of small droplets in the air above an orange orchard canopy during and after the sprayer operation. The authors of [38] developed a model to predict airborne drift based on the target structure. The model utilizes LIDAR measurements of optical transmission to predict the characteristics of airborne drift of PPPs leaving the target orchard at different growth stages and modified the drift characteristic for different methods of dose adjustment. Good agreement was demonstrated between the measurements and predictions of the drift from a semi-dwarf apple orchard at full-dose application rates.

**Figure 8.**
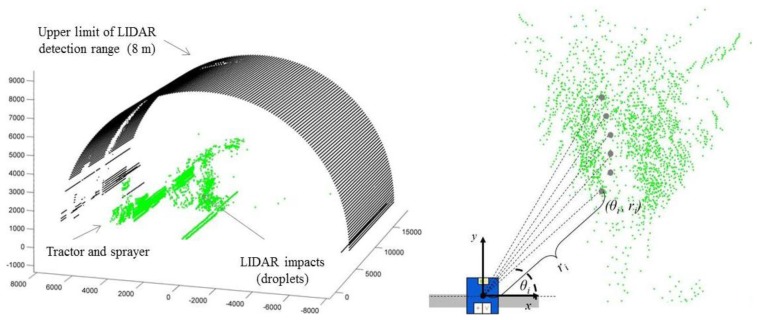
Example of LIDAR data plotted. 3D view of the drift cloud escaping the canopy (**Left**). Measurement process determining the values of angular position (*θ_i_*) and radial distance (*r_i_*) of every LIDAR impact (**Right**) (published in [7] with permission of the editor of Sensors journal).

Based on the above-described work, the research group arranged a research project [7] with the aims of (1) verifying the use of a LIDAR sensor to measure the drift cloud during pesticide application in a vineyard and (2) studying the effect of different working parameters (nozzle type, sprayer characteristics and air settings) on the total amount of liquid exceeding the target canopy. Drift measurements were made using a LIDAR sensor located at a distance 4 m from the last sprayed canopy row, oriented so that it could measure the cloud drift on a perpendicular plane relative to the canopy row (Figure 8).

The LIDAR scanner used in this work was a low-cost general-purpose Sick LMS-200, adjusted to have an angular resolution of 1° and a scanning angle of 180°. The spray drift cloud exceeding the canopy was scanned for an average of 40 s (the total time of LIDAR scanning in a single test) during the spray track along the row, for 20 s before the sprayer passed in front of the LIDAR and for 20 s afterward, representing a total measurement distance of 50 m. In order to compare the LIDAR measurements, spray deposit over 20 m away from the last row (perpendicular lane) was also collected and quantified using tartrazine as a tracer.

The obtained results indicate that, in general, the use of the LIDAR sensor represents an interesting and easy technique for establishing the potential drift of a specific set of sprayer settings and environmental conditions. The LIDAR system provides an idealized optical view of spray droplets escaping the canopy and its distribution in position with respect to the target. Furthermore, it allows drift to be evaluated with less labour, cost and time than other current methods. In general, a good correlation has been observed between the measured drift cloud with LIDAR and the deposition distribution obtained with the artificial collectors placed in the test bench. However, it seems that drift measurements using LIDAR can be affected by droplet size. The proposed use of LIDAR will help users to configure an adequate deposition over the whole canopy according to the specifications of the treatment and could be used as a drift prediction tool depending on the target geometry, also in accordance with [38].

As an alternative approach, the research group is developing a LIDAR-based system that analyses the temporal characteristics of the backscattered signal received when the laser beam passes through the drift cloud (range-resolved LIDAR) [40]. Such LIDAR devices, which are widely used in atmospheric studies, are based on different types of interaction between the electromagnetic radiation near the visible range and atmospheric molecules, elastic backscatter being the most commonly used technique. These systems allow the characterization and visualization, in near real-time, of the drift plume section in terms of the density of PPP droplets present in the air and hence allow the quantification of the amount of PPP lost. The LIDAR instrument being developed, specifically designed for the characterization of the PPP drift, benefits from the experience gained by the group as a result of various experimental trials with an ultraviolet LIDAR for atmospheric studies, which was employed to monitor PPP drift in orchards [41].

## 6. Software to Determine the Optimal Volume Rate in Vineyards

In the context of advanced technologies in vineyard spraying, several decision support systems (DSS) have been developed in the last decade for improving and making more sustainable pesticide applications (e.g., [42,43]). In this context, DOSAVIÑA [44,45] is a DSS used to determine the optimal volume rate for spraying of vineyards based on achieving the optimal coverage (impacts·cm^−2^) according to the characteristics of the crop canopy. It also considers the establishment of an efficiency factor that depends on aspects such as the type of sprayer used, nozzle type and size, operational parameters and weather conditions. Several field experiments showed that the use of this DSS reduced the volume rate by an average of 39.9% and reduced pesticide use by 53% in comparison with conventional applications, maintaining the deposition, uniformity and disease control [45]. DOSAVIÑA establishes the applied volume rate according to the characteristics of the canopy. This fact implies an important reduction in pesticide use in comparison with traditional application values. The results obtained demonstrated the amount of this reduction and also the corresponding application efficiency. Applications made according to the DOSAVIÑA recommendations resulted in generally higher values of recovery relative to the total applied amount that was retained on the leaves.

This reduction in pesticide use can be directly related to the intended global reduction in the use of pesticide proposed by the EU [37]. However, those values could also be linked to those proposed by [46], which stated that for agricultural applications, dose has little to do with efficacy because there is already sufficient pesticide to kill all the pests in the field many times over.

## 7. Conclusions/Outlook

All the previous research on the use of new technologies to improve the pesticide application process in viticulture has demonstrated interesting results in terms of the reduction of the total amount of pesticides used, reduction of the risk of environmental problems and the possibility to adapt the applied amount of PPP to the canopy characteristics. A further analysis of all those aspects, either individually or as a whole, indicates how all the new developments and their applications, in this case in viticulture, represent tools to implement the policies in the new and very restrictive European legal frame in all aspects concerning the use of plant protection products.

Since new legislation has applied efforts to the use-phase of pesticides, it is now time to integrate all the disposable tools that previous research have demonstrated to be interesting devices. However, there is still another key element that is absolutely needed to achieve success in the whole process: an adequate training of all the professionals involved in the process, which also according to the mandate established in [37] represents, as demonstrated by different researchers, the key factor for the whole integration. Only when adequate training has been achieved in all of the European territory will the system be able to implement the policies in the legal framework and to produce in a better and more sustainable way.
